# Paired Assessment of Volatile Anesthetic Concentrations with Synaptic Actions Recorded In Vitro

**DOI:** 10.1371/journal.pone.0003372

**Published:** 2008-10-08

**Authors:** Stuart J. McDougall, James H. Peters, Lia LaBrant, Xin Wang, Dennis R. Koop, Michael C. Andresen

**Affiliations:** 1 Department of Physiology & Pharmacology, Oregon Health & Science University, Portland, Oregon, United States of America; 2 BioAnalytical Shared Resource & PharmacoKinetics Core, Oregon Health & Science University, Portland, Oregon, United States of America; 3 Department of Pharmacology & Physiology, George Washington University, Washington, D. C., United States of America; Yale School of Medicine, United States of America

## Abstract

The volatile anesthetic isoflurane poses a number of experimental challenges in the laboratory. Due to its rapid evaporation, the open conditions of most in vitro electrophysiological recording systems make the determination of actual isoflurane concentrations a challenge. Since the absolute anesthetic concentration in solution is directly related to efficacy, concentration measurements are important to allow comparisons between laboratory and clinical studies. In this study we quantify the sources of isoflurane loss during experimentation and describe a method for the measurement of isoflurane concentrations using gas chromatography and mass spectrometry simultaneous to in vitro electrophysiological measurements. Serial samples of perfused bath solution allowed correlation of isoflurane concentrations with ongoing biological effects. Saturated physiological solutions contained 13.4±0.2 mM isoflurane and were diluted to desired “nominal” concentrations for experiments. The perfusion system established stable isoflurane concentrations within the bath by 2 minutes. However, bath isoflurane concentrations varied substantially and unpredictably between experiments. The magnitudes of such discrepancies in isoflurane concentrations spanned clinically important levels. Our studies suggest that, despite countermeasures, solution handling significantly impacted the isoflurane content in the tissue bath. The magnitude of these discrepancies appears to necessitate systematic direct measurement of bath isoflurane concentrations during most in vitro conditions.

## Introduction

Volatile general anesthetics, such as isoflurane, facilitate relatively rapid onset and recovery from anesthesia in the clinical setting. However, these compounds present unique challenges for in vitro studies of their biological effects under conditions in which experimental access to cells generally requires open conditions. In contrast to less volatile substances, loss of anesthetic from initial preparation to delivery to the tissue is a potential limitation during in vitro laboratory experiments. A calculated concentration of anesthetic mixed in solution can be prepared to a nominal concentration. However, in the course of an experiment, the low partition coefficient of a volatile anesthetic for aqueous solutions leads to phase separation and atmospheric loss from the solution. These processes often result in important discrepancies between nominal and actual drug concentrations in solution. In order to characterize biological actions, a physiological response is directly related to the concentration of the anesthetic during experiments. Thus, unknown or variable losses in volatile anesthetic content can lead to substantial underestimation of anesthetic efficacy.

Principally, two methods are commonly used to produce aqueous stock solutions of volatile anesthetics. In one method, the volatile anesthetic is added to a physiological solution and the two separate phases are allowed to equilibrate. Aliquots from the resulting saturated aqueous phase are then diluted to the desired nominal test concentrations and stored in an enclosed reservoir before delivery to the tissue bath, e.g. [1]. In the second method, a solution reservoir holding the physiological solution is bubbled to equilibration with vaporized isoflurane and concentrations are set to the desired nominal concentrations by a calibrated vaporizer, e.g. [2]. The detailed approaches to sampling from the resultant solutions, for either method, vary greatly across studies. Important differences between studies include the sites within the perfusion system from which samples are taken to measure anesthetic concentration (e.g. from the saturated source solution, the reservoir or the bath) and the times of measurement during a given experiment (e.g. before, during or after). Such pragmatic differences increase the difficulty of comparing results between studies.

As part of studies on the effects of isoflurane on glutamatergic and GABAergic neurotransmission to brainstem neurons [3], we implemented a serial sampling procedure of the bath solution coincident with our electrophysiological measurements. Our perfusion system delivered isoflurane in the range of 10 µM–1000 µM to brain slices in the bath. In our approach, small serial samples were taken from the bath at a site downstream from the brain slice and isoflurane concentrations were determined by gas chromatography/mass spectrometry (GC/MS) for correlation with measures of synaptic transmission. Our results suggest that, even with concerted efforts to control for procedural differences, nominal values overestimated measured isoflurane concentrations on a day-to-day basis by 10–75%. The magnitude of these differences suggests that measuring volatile anesthetics during in vitro experiments is required to assure accurate assessments of pharmacological results.

## Materials and Methods

All animal procedures were performed with the approval of the Institutional Animal Care and Use Committee at Oregon Health & Science University (Portland, Oregon) and conform to the guidelines described in the National Institutes of Health publication “Guide for the Care and Use of Laboratory Animals”.

### Preparing aqueous isoflurane solutions

For experiments on synaptic transmission in brain slices, 20 ml of isoflurane was added to 30 ml of physiologically formulated, artificial cerebrospinal fluid (ACSF) containing (mM): 125 NaCl, 3 KCl, 1.2 KH_2_PO_4_, 1.2 MgSO_4_, 25 NaHCO_3_, 10 glucose, and 2 CaCl_2_ bubbled with 95% O_2_/5% CO_2_ (300 mOsm). This 1∶1.5 v/v mixture was placed in a glass centrifuge tube and immediately capped with Teflon-lined cap and allowed to equilibrate at room temperature overnight. Samples taken from the upper ACSF phase contained 13.4±0.2 mM (n = 8) isoflurane (see below). Aliquots of the saturated ACSF stock were diluted in oxygenated ACSF for use during *in vitro* experiments to final nominal concentrations between 10 µM–1000 µM. In contrast, bubbling an ACSF/isoflurane solution (600 µM) with 95% O_2_/5% CO_2_ in an open container rapidly reduced isoflurane concentration to 50 µM after 20 min (data not shown).

### Measuring isoflurane concentration by GC/MS

GC/MS determination of isoflurane concentration (Hospira, Inc.; Lake Forest, IL) was based on modifications of a published protocol [4]. The experimental bath was sampled (100 µl) using gas-tight glass Hamilton syringes (Hamilton Company, Reno, NV). Samples were immediately deposited into ice cold glass vials containing n-heptane (500 µl, Mallinckrodt Baker, Inc.; Phillipsburg, NJ) and the internal standard, halothane (Halocarbon Laboratories; River Edge, NJ), then capped with Teflon-lined caps and kept on ice. Isoflurane was extracted into the organic phase by vortexing for 1 min. Vials were then centrifuged at 2000 rpm for 3 min to separate the phases. A 100 µl portion of the organic phase was transferred to autosampler vials (Sun-SRi, Rockwood, TN) and either processed immediately or stored at −80°C with standards prepared the same day. The standard isoflurane solutions were prepared by direct dilution with n-heptane. Standard curves with three isoflurane concentration ranges were used; 50–4000 µM isoflurane (bath dynamics, storage/shipping tests); 10–1000 µM (*in vitro* experiments, bath dynamics); and 1–100 µM (*in vitro* experiments). The internal standard (halothane) was varied with each range and contained 1000 µM, 500 µM and 50 µM, respectively. Isoflurane concentrations were determined from the area ratios of isoflurane/halothane compared to the standard calibration curves prepared from known amounts of isoflurane. On each day of experiments, bath samples were accompanied by a set of standards and processed similarly.

Analyses were performed using a Thermo Electron TRACE DSQ GC/MS system (Thermo Electron Corporation, Austin, TX) configured with an AS 2000 autosampler and a split/splitless injector. Isoflurane and halothane were separated on an AT-5ms fused silica capillary column (30 m×0.25 mm ID, 0.25 µm film thickness, Alltech Associates, Deerfield, IL) using helium as the carrier gas at a constant flow rate of 1 ml/min. Samples (1 µl) were injected in the split mode with a split rate of either 1∶100 or 1∶200 depending on the isoflurane concentration. The injector was maintained at 150°C and ion transfer line at 250°C. The TRACE GC Ultra oven was held constant at 40°C for 3 min and the temperature increased at 40°C/min to 120°C. The mass spectrometer was operated in the electron impact mode with an ionization energy of 70 eV and a source temperature of 250°C. Data were acquired in the selected ion monitoring mode with ions at *m/z* = 117 and 148 for isoflurane and *m/z* = 117 and 198 for halothane. Typical retention times were 1.61 min for isoflurane and 1.93 min for halothane. Instrument control and data acquisition and analysis were accomplished using Xcalibur Version 1.4 software (Thermo Scientific, Waltham, MA).

### Perfusion system for brain slices

Our standard perfusion apparatus for electrophysiological experiments was modified by the addition of 30 mL ground-glass syringes (BD Yale/BD Luer-Lok, Franklin Lakes, NJ) to serve as sealed reservoirs for the perfusate solutions. Reservoirs were connected to the bath via Teflon tubing (Small Parts Inc., Miami Lakes, FL). Reservoirs were filled completely to eliminate a headspace gas compartment within the sealed syringe. Reservoir syringes were mounted vertically in the perfusion apparatus with weights fixed onto the glass syringe plungers to provide a steady head pressure and facilitate flow. The flow rate was controlled with a fine needle valve (FR-55S, Warner Instrument Corporation, Hamden, CT) and maintained at 1.5 to 2 ml/min. At this flow rate the bath volume (0.5 ml) turned over a minimum of three times per minute. The surface area of the bath was approximately 2 cm^2^. Samples for isoflurane measurement were taken at fixed times and positions within the bath (see below).

### Whole cell recordings in brain slices

Brain stem slices were prepared from adult (>160 g) Sprague Dawley rats (Charles River Laboratories, Inc., Wilmington, MA) as described in detail previously [5]. Briefly, rats were deeply anesthetized with isoflurane and killed by cervical dislocation. The medulla was rapidly removed and was cut to yield a single 250 µm thick horizontal slice. The slices were secured to the floor of the tissue bath with a harp constructed of platinum wire and fine polyethylene strands and perfused with ACSF at 32–35°C.

Neurons in the solitary tract nucleus (NTS) were targeted for electrophysiological recordings [5]. Electrodes (3–4 MΩ) were visually guided to neurons using infrared illumination and differential interference contrast optics (40× water immersion lens) on an Axioskop-2 FS plus fixed stage microscope (Zeiss, Thornwood, NJ). The recording electrode solution contained (mM): 10 NaCl, 130 K-gluconate, 11 EGTA, 1 CaCl_2_, 1 MgCl_2_, 10 HEPES, 2 Na_2_ATP, and 0.2 Na_2_GTP at pH 7.3 and 296 mOsm. All recordings were made in open, whole-cell patch configuration under voltage clamp using an Axopatch 200A or Multiclamp 700B amplifier (Axon Instruments, Foster City, CA) and cells held at V_H_ = −60 mV. Signals were sampled at 30 kHz and filtered at 10 kHz using p-Clamp software (version 8.2, Axon Instruments, Foster City, CA). For data presented here, we assessed anesthetic actions on miniature inhibitory postsynaptic currents (mIPSCs) in second order medial NTS neurons [6] and were isolated by blockade of EPSCs with ionotropic glutamate receptor antagonists (2,3-dihydroxy-6-nitro-7-sulfonyl-benzo[f]quinoxaline, NBQX, 20 µM and D-2-amino-5-phosphonovalerate, AP-5; 100 µM) [7]. Miniature synaptic events were defined by the presence of tetrodotoxin (tetrodotoxin, TTX; 3 µM). A sensitive measure of anesthetic enhancement of GABA receptor function is the decay-time constant of the mIPSC [3], [8]. Events were detected and analyzed using MiniAnalysis (Synaptosoft, Decatur, GA) and decay-time constants were determined by fitting a single exponential between the 10% to 90% of peak amplitude portions of the current decay phase.

The isoflurane test solutions were delivered to the tissue bath via Teflon tubing and passed through a flow meter and heating element. The bath temperature was maintained between 32 and 35°C. For each isoflurane infusion, the bath was sampled at four time points: baseline, at 2 min before isoflurane; 2 min and 5 min during isoflurane and 2 min following the return to control bath solution. Slices were washed for at least 20 min following isoflurane exposure when subsequent concentrations were tested with the same slice preparation.

## Results

### Measuring isoflurane concentration in fresh and stored bath samples by GC/MS

**Figure 1:**
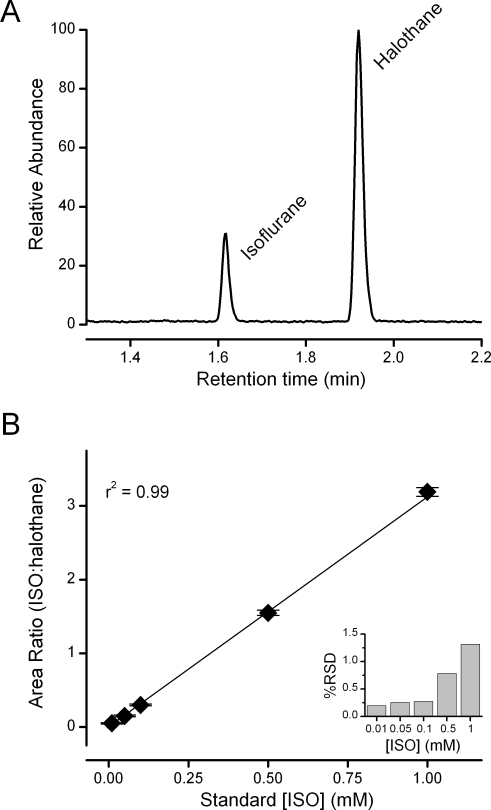
Isoflurane was reliably detected and measured by GC/MS. Bath samples (100 µl) were taken and isoflurane was extracted into a heptane/halothane solution, where halothane served as an internal control. A. A representative chromatogram demonstrating the detection of isoflurane and halothane at typical retention times. The area ratio of each peak was compared and allowed for precise determination of isoflurane concentration. On each experimental day bath samples were processed along side a set of standards. B. The average of 21 standard calibration curves used during *in vitro* experiments (values = mean±SEM). Standard isoflurane concentrations and the measured area ratio of isoflurane to halothane were highly correlated (R^2^ = 0.99). Insert; the greatest error between the curves occurred at 1000 µM isoflurane where the relative standard deviation (RSD) was 1.3%. The degree of accuracy in determining bath isoflurane concentration across experiments by GC/MS was very high.

**Figure 2:**
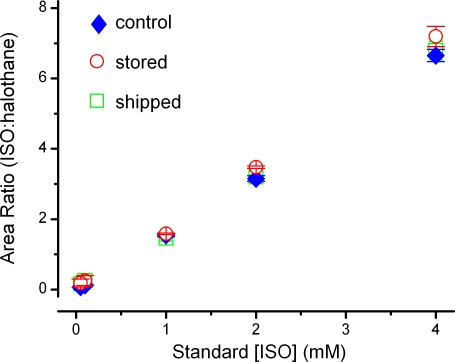
Standard curves were constructed over a concentration range of 50 µM–4000 µM isoflurane (control, closed blue diamonds, n = 4) to test the viability of sample storage at −80°C for 8 weeks (open red circles, n = 2) or shipped overnight on dry ice (open green squares, n = 5). These procedures, storage or shipping, had no significant effect on the accuracy of the standard curves particularly in the range of 0–1000 µM, which was used for *in vitro* experiments (p = 0.084 for group comparison and p = 0.808 for the interaction between the groups and standard concentrations by two-way ANOVA).

Isoflurane and halothane eluted in the GC/MS column at characteristic times with signal to noise ratios greater than 10∶1 (Figure 1A). The analytical method could reliably detect and quantify isoflurane well below minimal clinically effective concentrations. Inter-day variability was low with relative standard deviations ranging from 0.2 to 1.3% (Figure 1B, insert). To expand the practicality of collecting samples over a period of days, we assessed whether prolonged storage of extracted samples at −80°C or shipping extracted samples impacted the measurements. To test the stability of stored samples, solutions were prepared and extracted as described but stored for 8 weeks at −80°C before warming to room temperature before analysis. For the shipped samples, isoflurane solutions were prepared using identical protocols for production, sampling and extraction before being shipped overnight on dry ice from Washington, DC to Portland, Oregon where the GC/MS measurements were made. For these trials, standard curves ranging from 0–4000 µM isoflurane were used. This concentration range greatly exceeded that required for *in vitro* testing (10 µM–1000 µM), as we hypothesized that discrepancies might be exaggerated at high concentrations. The differences in handling time and procedures of these samples (stored vs. shipped) did not affect the standard curves (Figure 2, p = 0.084 for group comparison and p = 0.808 for the interaction between the groups and standard concentrations, two-way ANOVA).

### Delivery of isoflurane to the tissue bath

**Figure 3:**
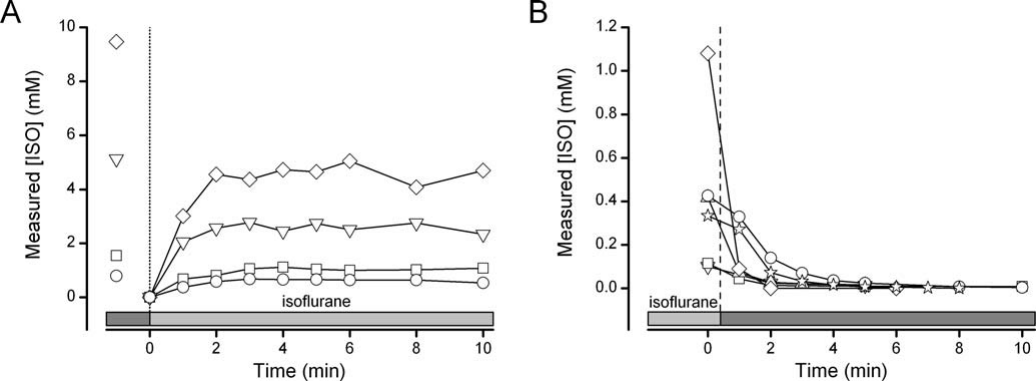
Isoflurane held in a sealed glass reservoir was plumbed to an *in vitro* tissue bath that was open to the atmosphere. A. In a representative experiment, measurements from the reservoir with ACSF saturated with isoflurane (diamond, 13.4 mM) or diluted; 1∶1 (triangle, nominal concentration 6.7 mM); 1∶3 (square, nominal concentration 3.35 mM) and 1∶9 (circle, nominal concentration 1.3 mM) were perfused through the bath and sampled at the bath outlet over time. There was a concentration dependent loss of isoflurane from the bath. Yet, isoflurane concentration in the tissue bath reached a steady state after ∼2 min (bath volume = 0.5 ml, flow rate 1.5–2 ml/min) B. Isoflurane cleared the bath within minutes when washed with ACSF at concentrations typically tested during *in vitro* electrophysiological studies.

Test solutions were prepared by mixing isoflurane with ACSF (1∶1.5 v/v) and this mixture was allowed to stand overnight in a gas-tight vessel. Following the equilibration, samples from the aqueous phase consistently contained 13.4±0.2 mM isoflurane (n = 8). This saturated solution of isoflurane in ACSF was then diluted to nominal concentrations and immediately transferred to a gas-tight reservoir. Samples taken for GC/MS measurements directly from the reservoirs were consistently less than the nominal concentrations calculated based on the dilution ratios (Figure 3A, far left points) and thus represent a preparative loss of anesthetic. In order to determine losses attributed to the delivery system and the time necessary to alter the bath concentration, we measured serial samples taken each minute following switching from a control solution (isoflurane-free) to perfusion with isoflurane test solutions (Figure 3). The concentration of isoflurane rose rapidly to steady levels at a fixed point within the tissue bath and these levels were maintained throughout the perfusion interval (Figure 3A). Generally, 2 min was sufficient time to reach a steady concentration at the standard flow rates for the electrophysiological experiments (Figure 3A). The bath isoflurane concentrations were substantially lower than those in the sealed reservoir and this difference represents the consistent loss of the volatile anesthetic from the delivery system and the bath (Figure 3A). As expected, isoflurane was lost at each stage of the protocol. Losses were incurred during the initial dilution of stock saturated isoflurane-ACSF and during the addition of the diluted stock to the reservoir. However, our interest focused foremost on the concentrations in solutions adjacent to the brain slices. From the initial saturated solution to the sampling in the final stage of the perfusion system, these trans-system losses were generally between 15 and 40%. For these tests (Figure 3A), we used the following solutions: ∼13.4 mM nominal, 1∶1 (∼6.7 mM nominal), 1∶3 (∼3.35 mM nominal) and 1∶9 (∼1.3 mM nominal). Despite the losses, steady levels in the perfusion bath were maintained so that measurements at 2 and 5 min during the electrophysiological studies accurately reflect brain slice exposures to isoflurane. Switching to control solution from test solutions resulted in a rapid disappearance of isoflurane from the bath at rates similar to bath loading (Figure 3B). Isoflurane was undetectable following 2–3 min of control perfusion so that allowance of 20 min wash periods in the electrophysiological studies offered an adequate margin of time for full recovery.

### Sampling during electrophysiological experiments

**Figure 4:**
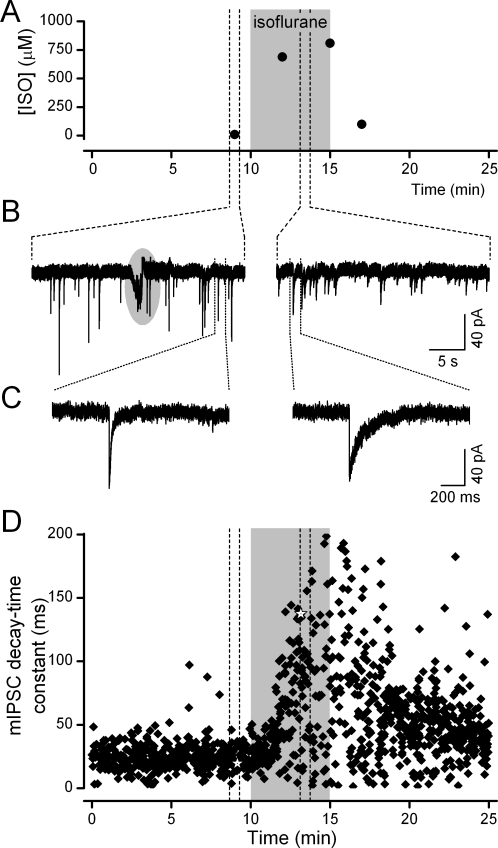
Bath samples (100 µL) were taken simultaneous to electrophysiological recordings to determine exact isoflurane concentrations. A. In this representative example, samples were taken from the bath 2 min before, 2 and 5 min during, and 2 min after isoflurane exposure. The measured values indicate a rapid isoflurane equilibration within the bath (∼2 min). Isoflurane was perfused for a total of 5 min (grey shading). B. A consequence of each sample taken from the bath was a temporary interruption of the electrophysiological recording (grey circular shading). This artifact provided a time stamp of when isoflurane measurements were taken. C. Individual miniature inhibitory post synaptic currents were elongated during isoflurane exposure at these concentrations. D. Isoflurane concentrations in the tissue bath, as determined by GC/MS, were precisely correlated with the increase in the decay-time constant of miniature inhibitory post synaptic currents (closed diamonds and open stars = events shown in C) over the course of the experiment.

During electrophysiological recordings (Figure 4), mIPSC characteristics closely corresponded to bath isoflurane concentrations. Using a gas-tight glass Hamilton syringe and needle, we sampled from the downstream outlet of the bath as a measure most closely approximating tissue exposure (Figure 4). Sampling produced a brief electrical artifact in the recordings that clearly delineated the sampling interval (Figure 4B). The arrival of isoflurane evoked a rapid increase in the mIPSC decay-time constant that was correlated with the bath concentration of isoflurane (Figures 4C & D). Following the return to control solution, the mIPSC time-decay constants rapidly returned to pre-isoflurane values (Figure 4D).

**Figure 5:**
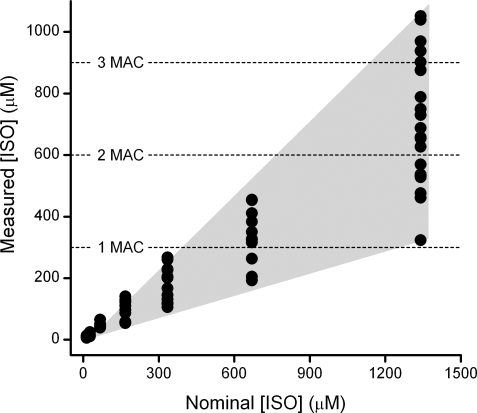
The concentration of isoflurane in the bath, as measured by GC/MS, is plotted against the nominal concentration of isoflurane achieved by dilution of ACSF saturated with isoflurane. For measured values, each point represents the average of two samples taken during each isoflurane exposure (at 2 and 5 min, Figure 4). The range of values (grey shading) at each nominal concentration indicates the variability of bath isoflurane concentrations. This loss occurred consistently despite identical procedures for diluting isoflurane to nominal concentrations for each experiment.

Our *in vitro* studies relied on the consistency of measured concentrations of isoflurane in saturated ACSF solutions as a starting point. We created a set of test solutions that contained nominal isoflurane concentrations calculated from the theoretical dilutions. This allowed us to target the range of concentrations of interest to the synaptic studies. A plot of these nominal isoflurane values across all trials versus the measured isoflurane bath concentrations (Figure 5) illustrates two important points. First, as expected, the average measured isoflurane bath concentrations were always lower than the nominal values due to cumulative isoflurane loss. Second, the bath concentrations varied greatly despite uniform handling and delivery. The degree of this variation corresponds to a range of concentrations that cross multiple clinical levels (1 to 3 MAC, mean alveolar concentrations) and thus could constitute a major limitation in studies in which isoflurane is not measured.

## Discussion

Here we demonstrate that a liquid/liquid extraction of isoflurane from a typical tissue bath can be conducted simply and effectively simultaneous with electrophysiological recordings. The concentration of isoflurane measured in an *in vitro* tissue bath closely corresponded to a physiological measure of volatile anesthetic action (mIPSC decay time constant). The sensitivity of the GC/MS measurements ensured accurate measurements and yielded high resolution concentration-response relationships. Despite rigorous procedural controls intended to consistently deliver the fixed concentrations of isoflurane, our measurements revealed inter-experiment variations in isoflurane delivery across a critical range of concentrations. We observed concentration-dependent losses of isoflurane at all stages of solution handling and delivery. Thus, the large discrepancy between the intended, nominal isoflurane concentration and the actual delivered concentration raises concerns about even well-controlled delivery protocols. Presumably the variability of these losses represents minor differences within procedures across trials and therefore is likely a measure of the pragmatic limits of such protocols. Our studies clearly identify the importance of measuring the final concentration of volatile anesthetic in the immediate vicinity of brain slices and that such measurements are critical to defining the biological mechanisms of action.

The accuracy and precision of isoflurane determination depend upon minimizing sampling errors within and between experiments. We attempted to control for errors in sample handling within experiments by the addition of an internal standard as a routine part of liquid/liquid extractions. The variance in the standard curves generated for each study offers a measure of the source of error between experiments. Our relative standard deviation was maximal at the high end of our standard curves (1 mM) and was only 1.3%.

Stock ACSF solutions saturated with isoflurane at room temperature contained 13.4±0.2 mM isoflurane and this corresponds to reports by others (10–15 mM) [1], [9]–[11]. The variation in saturated levels across these studies may be due to differences in the composition of these extracellular solutions. Loss of volatile anesthetics like isoflurane from a saturated aqueous solution is a certainty with handling (diluting, mixing, and perfusing). Such losses should be rise in proportion to the concentrations used due to increases in the partial pressure of isoflurane in solution. For example in our system, our highest, i.e. fully saturated, isoflurane-ACSF solution (13.4 mM) achieved a steady state bath level of ∼4.7 mM. Isoflurane likely evaporated during solution transfer to the sealed reservoir and subsequent perfusion of the tissue bath. We targeted a study range of 10–1000 µM isoflurane for our synaptic transmission studies. In this system evaporation of isoflurane from the bath was likely facilitated by heating to 32–35°C. Assessments of the effects of handling in other laboratories indicated similar trends in the loss of volatile anesthetics from aqueous solution. Diluting an aqueous solution saturated with isoflurane (15 mM) to 2000, 1000 or 500 µM (nominal) and then bubbling with O_2_/CO_2_ resulted in a final isoflurane concentration of 200–300 µM in the bath [10], [12]. In addition, a 20% loss (1000 µM to 800 µM) was noted at 25°C from reservoir to bath as measured by GC/MS [13]. Similarly, loss of drug between the reservoir and bath where the volatile anesthetic was vaporized into solution has been reported from 2% to 28% (isoflurane concentrations not noted) [2], [14].

Since losses of isoflurane from ACSF were inevitable, not surprisingly, we found that measured bath concentrations of isoflurane were lower than our nominal values calculated for our *in vitro* experiments. However the degree of variability amongst measured isoflurane samples was telling and cautionary. Our 1∶79 dilution of saturated isoflurane ACSF (168 µM nominal) resulted in bath concentrations that ranged from 54 to 129 µM (>2 fold discrepancy). At the highest concentrations used, the 1∶9 dilution of saturated isoflurane ACSF (1340 µM nominal) resulted in bath concentrations ranging from 323 to 1051 µM (>3 fold discrepancy). Clearly, an analysis of the biological effects based on nominal isoflurane concentrations would have not only erred in the absolute concentration (much less) but also resulted in a high degree of error across day to day measures. Our studies offer a measure of the magnitude of this error that in turn contributes to the variability of the biological effects expressed in concentration response relationships and estimates of functional potency.

A possible alternative to intermittent sampling of the bath perfusate uses calcium sensitive electrodes to monitor bath concentrations continuously but relies on a indirect measure [15]. Measures with these electrodes are influenced by pH, osmolarity and composition of the external solution [15]. These requirements are normally well controlled during any electrophysiological study. Measures with these electrodes for anesthetic detection have standard deviations of ∼20% at 1 MAC isoflurane and 35°C [15] and such precision and sensitivity may limit their applications. Calcium sensitive electrode detection of general anesthetics is particularly well suited to ensuring delivery of stable isoflurane concentrations [16].

To assess current anesthetic pharmacological practices for comparison, the published reports in the PubMed database were surveyed by searching the first 7 months of 2008 using the terms “isoflurane” and “2008”. These terms yielded 419 hits. This pool of publications was narrowed by eliminating all clinical and whole animal studies to focus on *in vitro* experiments conducted using bath applications. Each article of the resulting 21 *in vitro* studies was read to note both the methods of measuring anesthetic bath concentration and whether the results of those measurements were reported. Two-thirds of these reports in the Methods sections indicated that bath or wells were sampled for GC measurement of isoflurane concentration. However, only four articles reported actual measured concentrations as an explicit result and two of these cases reported only an aggregate mean isoflurane concentration–essentially averaging over daily measurements. The remaining two cases included our study[3] and did report bath concentrations that were linked to individual trials of measured biological effects and thus tried to draw paired comparisons of cause and effect rather than unlinked correlates. In one-third of the articles, no mention was made of measurements of isoflurane concentration within the course of their report even though several referred to earlier or pilot studies as representing assessments of their nominal mixtures of isoflurane. Thus, most reports relied solely on nominal concentrations to interpret their findings. Often gas phase concentrations were monitored by infrared gas analysis, but recent reports suggest that this approach may suffer importantly in accuracy (+/−20%) compared to GC [17]. Temperature varied widely across these preparations despite its impact on the equilibration and biological actions of volatile anesthetics [18]. This literature survey suggests that despite advances in instrumentation and rigorous experimental practices, the importance of the daily variation in delivered volatile anesthetic is rarely reflected in reporting assessments of their biological effects.

Our studies demonstrate that direct sampling plus the sensitivity and accuracy of GC/MS measurements offer distinct advantages for the study of volatile anesthetics and understanding the variability of biological responses. Ease of setup, operational simplicity, high precision and reliable storage of samples are all pragmatic bonuses. Sampling at the level of the tissue from the bath during electrophysiological recordings did require a steady hand. Yet, GC/MS offers a rigorous and high degree of measurement resolution in the range of the isoflurane dose response curve of most interest, ≤300 µM for isoflurane near its calculated EC_50_ for anesthesia [19]. Accurate measures of anesthetic concentrations in *in vitro* bath solutions are essential in *in vitro* experiments to assure the translation of mechanistic observations to the clinical setting [20].
